# Gibberellin-to-abscisic acid balances govern development and differentiation of the nucellar projection of barley grains

**DOI:** 10.1093/jxb/eru289

**Published:** 2014-07-14

**Authors:** Diana Weier, Johannes Thiel, Stefan Kohl, Danuše Tarkowská, Miroslav Strnad, Sara Schaarschmidt, Winfriede Weschke, Hans Weber, Bettina Hause

**Affiliations:** ^1^Leibniz-Institut für Pflanzengenetik und Kulturpflanzenforschung, D-06466 Gatersleben, Germany; ^2^Leibniz-Institut für Pflanzenbiochemie, D-06120 Halle (Saale), Germany; ^3^Laboratory of Growth Regulators, Palacky University and Institute of Experimental Botany, Academy of Sciences of the Czech Republic, Slechtitelu 11, CZ-78371, Olomouc, Czech Republic; * Present address: Humboldt-Universität zu Berlin, Faculty of Agriculture and Horticulture, D-14195 Berlin, Germany.

**Keywords:** Assimilate transfer, barley endosperm, gibberellin-to-abscisic acid balances, maternal–filial communication, nucellar projection, *seg*8 barley mutant.

## Abstract

In cereal grains, the maternal nucellar projection (NP) constitutes the link to the filial organs, forming a transfer path for assimilates and signals towards the endosperm. At transition to the storage phase, the NP of barley (*Hordeum vulgare*) undergoes dynamic and regulated differentiation forming a characteristic pattern of proliferating, elongating, and disintegrating cells. Immunolocalization revealed that abscisic acid (ABA) is abundant in early non-elongated but not in differentiated NP cells. In the maternally affected shrunken-endosperm mutant *seg8*, NP cells did not elongate and ABA remained abundant. The amounts of the bioactive forms of gibberellins (GAs) as well as their biosynthetic precursors were strongly and transiently increased in wild-type caryopses during the transition and early storage phases. In *seg8*, this increase was delayed and less pronounced together with deregulated gene expression of specific ABA and GA biosynthetic genes. We concluded that differentiation of the barley NP is driven by a distinct and specific shift from lower to higher GA:ABA ratios and that the spatial–temporal change of GA:ABA balances is required to form the differentiation gradient, which is a prerequisite for ordered transfer processes through the NP. Deregulated ABA:GA balances in *seg8* impair the differentiation of the NP and potentially compromise transfer of signals and assimilates, resulting in aberrant endosperm growth. These results highlight the impact of hormonal balances on the proper release of assimilates from maternal to filial organs and provide new insights into maternal effects on endosperm differentiation and growth of barley grains.

## Summary

Hormonal balances of abscisic acid-to-gibberellic acid govern the development and differentiation of the nucellar projection, the maternal organ of barley grains involved in assimilate transfer and endosperm growth.

## Introduction

The nucellar projection (NP) develops from the nucellus tissue facing the main vascular bundle and reveals a complex pattern of simultaneous cell division, differentiation, and disintegration (Thiel *et al.*, 2008; Radchuk *et al.*, 2011). The release of assimilates from the nucellus and the NP partially depends on programmed cell death (PCD) (Radchuk *et al.*, 2006). Hence, the NP represents an important interface, which accomplishes transfer and inter-conversion of assimilates (Thiel *et al.*, 2009), and generates and transmits signals required for regulated development of filial tissues. In such a way, proper differentiation of the NP is tightly coordinated with that of endosperm. The process underlies hormonal regulation, and transcriptome analysis has revealed that gibberellins (GAs) participate to establish the differentiation gradient within the NP (Thiel *et al.*, 2008). The development of a robust NP is restricted to the Triticeae and is regarded as a key feature in domestication towards the selection for larger and round grains. Lack of a robust NP as in the Brachypodieae and Bromeae might explain their flat and starch-poor grains (Hands *et al.*, 2012). Thus, analysing the development of the NP addresses important yield-related traits.

In barley, endosperm cellularization starts 3–4 d after fertilization (DAF) and is completed within 1–2 d. Cell differentiation is initiated at the maternal–filial boundary starting within the outermost cell row adjacent to the NP and first generates the endosperm transfer cells. At 10 DAF, the endosperm begins to accumulate storage products. The pre-storage phase from anthesis to 4 DAF and the storage phase from 10 DAF are separated by a transition stage characterized by transcriptional reprogramming promoting the switch of the endosperm into the storage mode (Sreenivasulu *et al.*, 2004). Desiccation starts at physiological maturity after 20 DAF and grains reach full maturity at around 40 DAF.

Seed growth and development are regulated by phytohormones (Kucera *et al.*, 2005). The levels of GAs and abscisic acid (ABA) are negatively correlated and fluctuate during seed development, implicating a tightly regulated balance between these hormones (Batge *et al.*, 1999; White *et al.*, 2000). In general, GAs stimulate growth by cell elongation and can promote developmental timing and switches (Gazzarrini *et al.*, 2004; Hedden and Thomas, 2012). ABA functions antagonistically to GA and generally inhibits growth and cell elongation at higher concentrations but is also required for seed maturation events such as sugar signalling, storage product accumulation, desiccation, stress tolerance, and seed dormancy (Finkelstein and Gibson, 2002; Weber *et al.*, 2005). The mutual antagonism between GAs and ABA governs the decision between precocious germination or quiescence and maturation in cereals (White *et al.*, 2000; Jacobsen *et al.*, 2002). In tobacco seeds, GA stimulates the growth potential of the embryo by inducing cell-wall hydrolases. ABA represses these hydrolases and thereby endosperm weakening and embryo growth (Leubner-Metzger, 2002).

The *seg8* mutant is a recessive shrunken barley endosperm mutant, whose phenotype is dependent on the maternal genotype and is visible only in the endosperm (Felker *et al.*, 1985). The endosperm of *seg8* has only 27% of grain weight of the wild type (Röder *et al.*, 2006). The *seg8* endosperm cellularizes abnormally. Disturbed cell proliferation within the dorsal endosperm opposite the NP causes shrinkage of central parts of the *seg8* endosperm. Transfer cells, aleurone, and subaleurone cells are absent or substantially reduced, but differentiation is barely changed within the lobe areas. The number of starchy endosperm cells is strongly decreased due to the absence or a reduced number of starchy endosperm prismatic cells (Sreenivasulu *et al.*, 2010; Melkus *et al.*, 2011). Due to the underdeveloped endosperm, *seg8* grains adopt a characteristic flattened shape.

Impaired development of the *seg8* endosperm may be derived from a deregulated ABA signal. The levels of ABA are lower during the pre-storage and higher during the transition stage from cell division/differentiation to storage product accumulation. Basal levels of ABA, which do not induce stress responses, can promote growth (Chen *et al.*, 2003; LeNoble *et al.*, 2004; Barrero *et al.*, 2005). Insufficient ABA amounts may cause disturbed cellularization of the early *seg8* endosperm due to disturbed cell-cycle regulation, especially in regions where transfer cell differentiation is initiated (Sreenivasulu *et al.*, 2010). Similarly, in tobacco, maternal ABA, synthesized in seed coats, is translocated to the seed, promoting early seed development and growth (Frey *et al.*, 2004). Thus, in *seg8*, ABA transfer from vegetative into filial tissues could be prevented between anthesis and cellularization and the *seg8* phenotype may partially be caused by a disturbed ABA-releasing pathway (Sreenivasulu *et al.*, 2010).

The primary gene defect in *seg8* is so far unknown but must lie within maternal grain organs. Thus, altered development of *seg8* endosperm is probably elicited by aberrations within the maternal grain tissue. In particular, the NP could well be involved as it represents the interface between maternal and filial grain tissues and the site where assimilates and signals are transferred from the maternal to the filial organs. Our analysis of the development of NPs in barley grains revealed that differentiation is driven by a developmentally regulated spatio-temporal shift from lower to higher GA:ABA ratios. Deregulated GA:ABA balances, as in *seg8*, impair differentiation of the NP, potentially compromise transfer of signals and assimilates, and cause aberrant endosperm growth. Our results highlight the importance of GA:ABA balances for maternal effects on endosperm growth and differentiation to guarantee proper assimilate transfer.

## Material and methods

### Plant material

*Hordeum vulgare* L. var. Bowman *seg8* and *Hordeum vulgare* L. var. Bowman were obtained from J.D. Franckowiak (North Dakota State University, Fargo, ND, USA). *seg8* was identified as a spontaneous mutant in line 60Ab1810-53, later released as the cultivar Klages (Ramage and Crandall, 1981). The original mutant was backcrossed four times to cultivar Bowman (J.D. Franckowiak, personal communication). *seg8* and Bowman plants were grown in greenhouses under long-day conditions (16/8h light/dark at 19/14 °C) during spike and grain development. Flowering and developmental stages were determined (Weschke *et al.*, 2000). Seeds from different stages were harvested and snap frozen in liquid nitrogen for hormone measurement or fixed immediately for histology. For antibody tests, segments of 5cm length of primary leaves of 7-d-old Bowman seedlings were used after infiltration with 100 µM ABA.

### Quantitative determination of ABA and GAs

Samples were analysed for GA content according to Urbanová *et al*. (2013) with modifications. Seed samples (50mg dry weight) were homogenized in 2ml polypropylene tubes with 1ml of 80% (v/v) acetonitrile containing 5% (v/v) formic acid and 19 internal GA standards ([^2^H_2_]GA_1_, [^2^H_2_]GA_3_, [^2^H_2_]GA_4_, [^2^H_2_]GA_5_, [^2^H_2_]GA_6_, [^2^H_2_]GA_7_, [^2^H_2_]GA_8_, [^2^H_2_]GA_9_, [^2^H_2_]GA_12_, [^2^H_2_]GA_12_ald, [^2^H_2_]GA_15_, [^2^H_2_]GA_19_, [^2^H_2_]GA_20_, [^2^H_2_]GA_24_, [^2^H_2_]GA_29_, [^2^H_2_]GA_34_, [^2^H_2_]GA_44_, [^2^H_2_]GA_51_ and [^2^H_2_]GA_53_) (OlChemIm, Olomouc, Czech Republic) using an MM 301 mixer mill (Retsch, http://www.retsch.com) at a frequency of 27 Hz for 3min after adding 2mm zirconium oxide beads to each tube to increase the extraction efficiency. The tubes were then placed in a 4 °C fridge and extracted overnight with constant stirring at a frequency of 15rpm. The homogenates were centrifuged for 10min at 4 °C. Supernatants were further purified using mixed-mode anion exchange cartridges (Waters, http://www.waters.com) and analysed by ultrahigh-performance chromatography (Acquity UPLC™ System; Waters) coupled to triple-stage quadrupole mass spectrometer (Xevo^®^ TQ MS; Waters) equipped with an electrospray ionization (ESI) interface. GAs were detected using the multiple-reaction monitoring mode based on transition of the precursor ion [M-H]^–^ to the appropriate product ion. Data were acquired and processed by Masslynx 4.1 software (Waters) and GA levels were calculated using the standard isotope-dilution method (Rittenberg and Foster, 1940).

ABA was extracted and analysed according to Balcke *et al.* (2012). Briefly, 20–50mg of fresh material was homogenized in a mortar under liquid nitrogen and extracted with 500 μl of methanol containing 0.1ng μl^–1^ of isotope-labelled internal standard ^2^H_6_-ABA using a bead mill (FastPrep24; MP Biomedicals, http://www.mpbio.com). After centrifugation, 450 μl of supernatant was diluted with distilled water to 5ml and subjected to solid-phase extraction, performed in a 96-well plate format using filter plates (Agilent Technologies, http://www.agilent.com) packed with 50mg of strong cation exchange HR-XC material (Macherey–Nagel, http://www.mn-net.com) and deep-well receiving plates in conjunction with centrifugation. The material was conditioned with 1ml of methanol and equilibrated with 1ml of distilled water. Plant extracts were loaded in each well and fractions containing phytohormones were eluted with 900 μl of acetonitrile. Separation using the ACQUITY UPLC System (Waters) and detection by ESI-tandem mass spectrometry (MS/MS) using 3200 Q TRAP^®^ LC/MS/MS mass spectrometer (Waters) was performed as described previously (Balcke *et al.*, 2012).

### Generation of antibodies against ABA

ABA was coupled to BSA using 1-ethyl-3-(3-dimethylaminopropyl) carbodiimide (EDC; Merck, http://www.merck.com) and purified by dialysis against 0.1M sodium borate (pH 8.5). ABA–BSA was used to immunize two rabbits as described previously (Mielke *et al.*, 2011). Both sera showed strong binding of ABA–BSA and free ABA as tested by ELISA. Sera were tested by competitive ELISA according to Mielke *et al.* (2011) revealing specific ABA binding. Other hormones, such as jasmonic acid, could not compete with ABA for binding (Supplementary Fig. 1A at *JXB* online).

### Immunocytochemistry and histological analyses

Small pieces of plant material were fixed with 4% (w/v) EDC in PBS and embedded in polyethylene glycol 1500 (Merck) for immunocytochemical analyses (Mielke *et al.*, 2011). Ethanol (50%, v/v), 5% (v/v) acetic acid, and 3.7% (w/v) formaldehyde were used to fix material for nucleic acid staining and terminal deoxynucleotidyl transferase dUTP nick end labelling (TUNEL) experiments followed by paraffin embedding. Semi-automated immunolabelling experiments were performed using InSituPro VSi robot (Intavis, http://www.intavis.com) following protocols suggested by the manufacturer. Cross-sections (3 μm thickness) of EDC-fixed material were immunolabelled with the anti-ABA antibody diluted 1:10 000 in PBS containing 5% (w/v) BSA. As secondary antibody, goat anti-rabbit IgG was used conjugated with Alexa Fluor 488 (Invitrogen, http://www.lifetechnologies.com) or alkaline phosphatase (Merck) diluted 1:1000 in PBS.

Cell death detection was done in cross-sections (12 μm thickness) of formaldehyde/acetic acid/ethanol-fixed seed material by a TUNEL assay (Radchuk *et al.*, 2011). Cross-sections of the same material were used to stain nucleic acids using acridine orange. After removing the paraplast and rehydration, sections were washed in 0.2M acetate buffer (pH 2.1), stained with 0.05% (w/v) acridine orange, and washed twice in veronal-acetate buffer (pH 7.8). Fluorescence was analysed using excitation with blue light. Epifluorescence and light microscopy was done using a Zeiss ‘AxioImager’ microscope equipped with an AxioCam (Zeiss, http://www.zeiss.com). Micrographs were processed through Photoshop CS3 software (Adobe Systems, http://www.adobe.com).

### Microdissection of the NP, RNA extraction, and quantitative reverse transcriptase (qRT)-PCR

Caryopses of Bowman and *seg8* were harvested at 5, 7, and 10 DAF, frozen in liquid nitrogen and transferred to a cryostat (20 °C). The middle parts of caryopses were cut by razor blade and glued onto a sample plate using Tissue-Tek® O.C.T™ compound (Sakura Finetek Europe, http://www.sakuraeu.com). Sections of 20 µm were mounted in the cryostat chamber on membrane slides (MMI, http://www.molecular-machines.com) and stored for 7 d in the cryostat at –20 °C until complete dryness. Prior to microdissection, dry cryosections were adapted to room temperature.

Laser microdissection-assisted isolation of cells of NPs was conducted using a CellCut system (MMI). Total RNA was extracted from 20–30 sections per sample as described previously (Thiel *et al.*, 2011) and reversely transcribed into cDNA using SuperScript III (Invitrogen). Reactions were performed with Power SYBR Green PCR Mastermix (Applied Biosystems, http://www.appliedbiosystems.com) in a 7900 HT real-time PCR system (Applied Biosystems). Five biological replications were conducted for each gene. *HvActin1* (GenBank accession no. AK365182) was used for normalization of target genes as it was validated as suitable reference gene for qRT-PCR analysis in isolated barley grain tissues (Thiel *et al.*, 2008). Actin genes have also been shown to be stably expressed during seed development or in different *Brassica* seed tissues by a cross-species analysis, despite other superior reference genes being identified (Graeber *et al.*, 2011). Values were calculated as arithmetic means of the replicates and given as relative expression (1+E)(–ΔCt) according to Czechowski *et al.* (2004). Dissociation curves confirmed the presence of single amplicons in each reaction. The efficiencies of PCRs were determined using LinRegPCR software (http://www.gene-quantification.com/download.html). Only reactions with a PCR efficiency between 1.8 and 2.0 and a correlation of standard curves >0.995 were used for calculations.

### Accession numbers

For 9-*cis*-epoxycarotenoid dioxygenases (NCEDs): HvNCED1, BAF02837.1; HvNCED2, BAF02838.1; HvNCED3, BAK03427.1; HvNCED6, CAJX010148304.1. For aldehyde oxidases (AAOs): HvA AO1, BAK02080.1; HvAAO2, BAJ91458.1; HvAAO3, AK253133.1; HvAAO42, CAJX010121362.1; HvAAO5, AK252728.1; HvAAO6, BAJ89572.1. For GA20 oxidases (GA20ox): HvGA20ox1, AAT49 058.1; HvGA20ox21, CAJW010040861.1; HvGA20ox3, AAT490 59.1; HvGA20ox4, BAK04752.1; HvGA20ox5, BAK04700.1; HvG A20ox6, BAJ86897.1. For GA2 oxidase (GA2ox): HvGA2ox12, CAJ W010060897.1; HvGA2ox22, CAJX010058436.1; HvGA2ox3, BAJ9 5978.1; HvGA2ox4, AAT49062.1; HvGA2ox5, AAT49063.1; HvGA 2ox6, BAJ88432.1; HvGA2ox7, BAJ87891.1, HvGA2ox8, BAJ9 2832.1. For GA3 oxidase (GA3ox): HvGA3ox1, AAT49060.1; HvGA 3ox2, AAT49061.1.

## Results

**Fig. 1.**
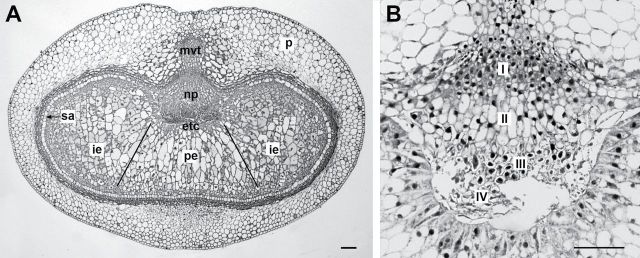
Light micrographs showing median cross-sections through wild-type barley grain at 8 DAF. (A) General view on tissues and organization of the barley grain. p, Pericarp; np, nucellar projection; etc, endospermal transfer cells; pe, prismatic endosperm; ie, irregular endosperm; sa, subaleurone. (B) Detailed view of the NP with morphologically different cell types. I, meristematic isodiametric cells; II, elongated cells; III, cuboid cells with thickened cell walls; IV, autolysing cells with wall ingrowths. Bars, 100 µm.

During barley grain development, assimilates are transferred from the main vascular bundle via NP and endosperm transfer cells into the endosperm (Fig. 1A). Thereby, the NP changes developmentally and consists of four main cell types (Fig. 1). The cells of the NP display cell division, differentiation, and disintegration (Linnestad *et al.*, 1998; Thiel *et al.*, 2008). Adjacent to the crease vascular bundle, NP cells are meristematic and isodiametric (type I in Fig. 1B). Cells in the mid-zone are elongated and differentiated (type II). Cubical cells with thickened cell walls occur adjacent to the endosperm (type III). The cells adjacent to the endosperm transfer cells develop wall ingrowths and are autolysing (type IV) at the end of the transition stage. Disintegration by autolysis of these cells is accompanied by PCD (Radchuk *et al.*, 2011) and generates the endosperm cavity. The structural framework of type III and type IV cells is possibly involved in assimilate transfer. The starchy endosperm contains two cell types, irregular cells within the lobes and prismatic cells between crease area and dorsal side of the caryopsis (Fig. 1A).

### Developing *seg8* grains are morphologically different from Bowman

Grains of *seg8* displayed a shrunken endosperm. From the side view, mature *seg8* grains appeared bulgy in basal parts and flattened in apical regions (Supplementary Fig. 2 at *JXB* online). Compared with Bowman, cellularization and starch accumulation in *seg8* endosperm started 1 d later (Supplementary Fig. 3 at *JXB* online). Most obviously, the NP was attached to the dorsal nucellar epidermis and integument region during the transition phase of *seg8*. The prismatic endosperm failed to develop. From 10 DAF onwards, the prismatic endosperm started to develop in the basal part of the *seg8* caryopsis (Supplementary Fig. 3).

**Fig. 2.**
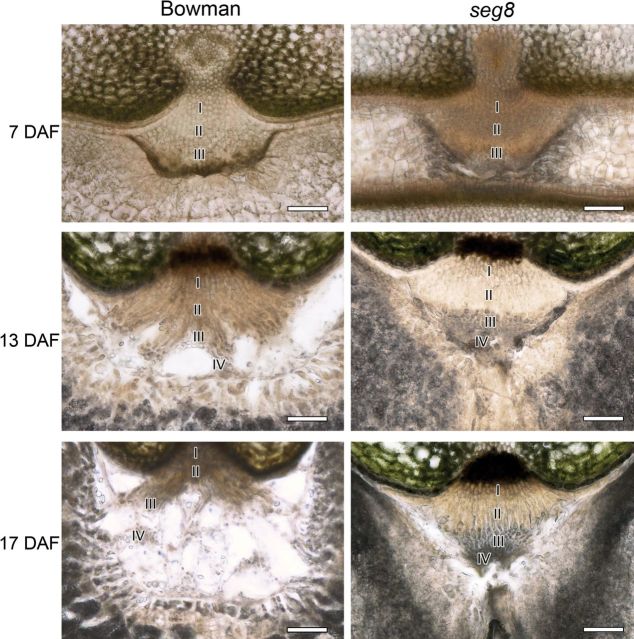
Cross-sections showing differences in NPs of Bowman and *seg8*. Different cell types of the NP are labelled. Note that in *seg8* the NP adjoins the dorsal site (7 and 17 DAF). The *seg8* NP maintains its compact shape during the whole development, type IV cells are not present, and the endosperm cavity is absent. Cell types I–IV are described in Fig. 1. Bars, 100 µm.

In Bowman, the NP altered its shape during grain development (Fig. 2). It appeared roundish and compact at pre-storage and transition phases. During the storage phase, NP cells disintegrated and an endosperm cavity was formed by autolysis of type IV cells. The NP of *seg8* developed differently compared with Bowman. Cross-sections showed changes in the *seg8* NP during grain development with labelled cell types (Fig. 2). The NP of *seg8* appeared compact during the whole development. The endospermal cavity and the structural framework of autolysing cells were absent. Because of the missing prismatic endosperm, a crease was formed from the dorsal site dividing the *seg8* endosperm into two lobes (Supplementary Figs 2 and 3). In regions without prismatic endosperm, the NP adjoined the dorsal side of the nucellar epidermis.

### Cell division, cell elongation, and PCD in the NP

**Fig. 3.**
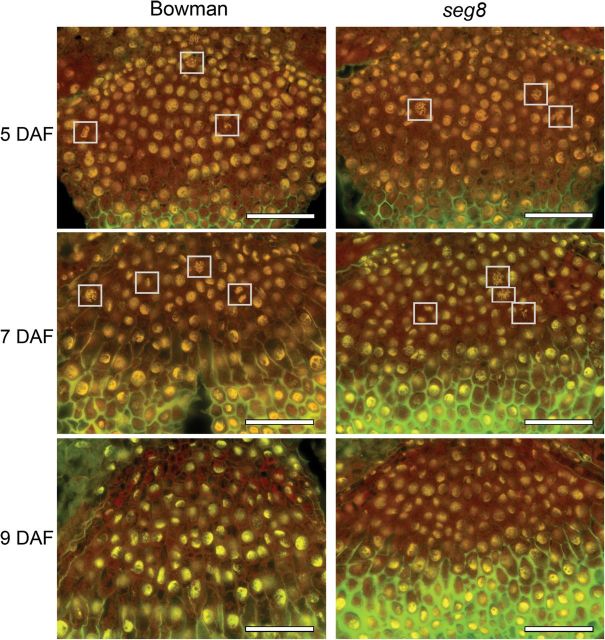
Cross-sections through developing NPs of *seg8* and Bowman stained with acridine orange. Red, RNA; yellow, DNA; green, autofluorescence of cell walls. Note the cell divisions evidenced by loss of typical nuclei, but condensed chromosomes (white squares). Dividing cells were visible in type I cells of Bowman and *seg8* up to 7 DAF. From 8 DAF, cell divisions were not visible. Bars, 100 µm.

Nucleic acids can be selectively stained by acridine orange. Red colouring indicates the presence of RNA and yellow indicates DNA. The staining exposes mitotic-active cells by labelling of condensed chromosomes, thus showing cell-cycle activity. In *seg8* and Bowman NPs, mitotic-active nuclei were present at similar numbers at the transition phase, at 5 and at 7 DAF (Fig. 3). Per cross-section analysed (*n*≥6), the NPs of Bowman showed 3.5±0.7 and 3.8±0.8 mitotic cells at 5 and 7 DAF, respectively. The NPs of *seg8* showed 3.8±1.2 and 3.6±0.5 mitotic cells at 5 and 7 DAF, respectively, which were not significantly different from the NPs of Bowman. In both genotypes, cell divisions were absent at 9 DAF (Fig. 3). Similar cell-cycle activities of *seg8* and Bowman NPs indicated that cell divisions in *seg8* NPs were not affected and probably not involved in developmental aberrations.

**Fig. 4.**
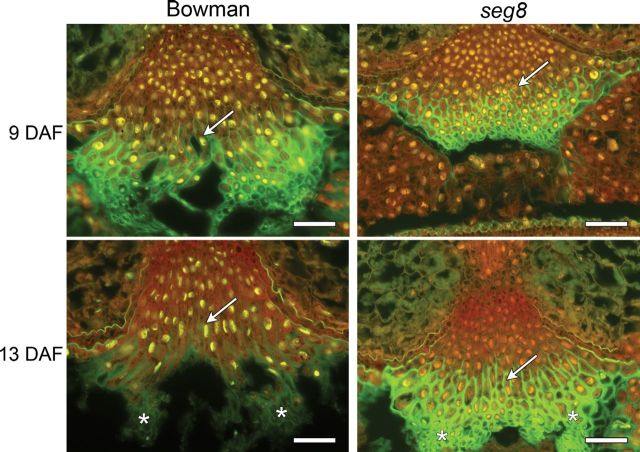
Cross-sections through NPs of *seg8* and Bowman stained with acridine orange. Red, RNA; yellow, DNA; green, autofluorescence of cell walls. Note the differences in cell elongation of type II cells of both genotypes at 9 DAF (white arrows). At 13 DAF, type II cells of the Bowman NP were clearly elongated, but only minor cell elongation was visible in the NP of *seg8*. Moreover, strong autofluorescence of cell walls was seen at 13 DAF in type III cells of *seg8* but not in the Bowman NP (white arrow). In *seg8*, autolysing type IV cells normally visible in Bowman and characterized by missing stainable RNA were replaced by small round cells with thickened walls (asterisks). Bars, 100 µm.

However, cell elongation within type II cells was found to be altered in *seg8* NPs (Figs 3 and 4). At the transition stage, at 7 and 9 DAF, these cells were not elongated in contrast to Bowman (Fig. 4, white arrows). In Bowman caryopses at 9 DAF, the average length of type II cells was 65.2±17.3 µm, whereas the same cells exhibited a length of 21.7±8.8 µm in caryopses of *seg8*, which was significantly different (*P*<0.01 according to Student’s *t*-test, *n*≥25 from at least four different sections). During the early storage phase at 13 DAF, only slightly elongated type II cells were visible in *seg8*. Autolysing cells were not present in *seg8* NPs but were numerously present in Bowman NPs (Fig. 4, asterisks). In *seg8* NPs, strong autofluorescence was observed within type III cells at 13 DAF (Fig. 4, white arrow), indicating thickened cell walls, which were absent in Bowman. This suggested that autolysis does not occur in *seg8* NPs and that cells with thickened walls are maintained.

**Fig. 5.**
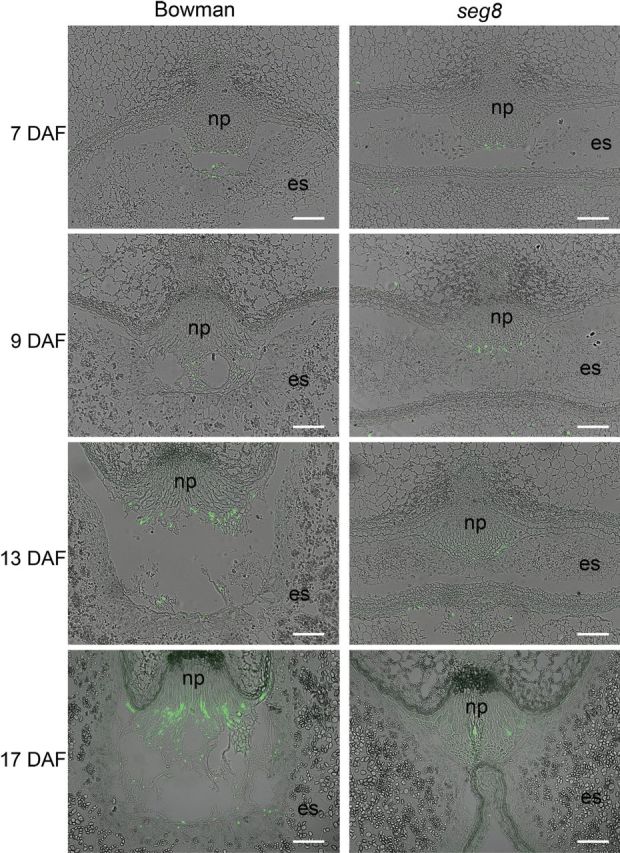
PCD in regions of type IV cells of NPs of Bowman and *seg8* shown by a TUNEL assay. Note that PCD events (green labelling) increased in Bowman but not in *seg8* during NP development. np, Nucellar projection; es, endosperm. Bars, 100 µm.

Cell disintegration within the NP is coupled to PCD (Radchuk *et al.*, 2011), indicated by degradation of nuclear DNA. The resulting DNA fragments can be cytochemically detected by a TUNEL assay of the 3′OH groups. In Bowman NPs, PCD events increased during transition and were most strongly pronounced in type IV cells at storage phase at 17 DAF (Fig. 5). This contrasted with *seg8* NPs, where the TUNEL assay revealed that PCD events declined during development from 7 DAF onwards and were nearly undetectable during the storage phase at 17 DAF. This result revealed no PCD detectable during the storage phase in *seg8* NPs, indicating that cell disintegration was absent here.

### Localization and distribution of ABA in the NP

In the filial fraction of *seg8* grains containing the NP by co-isolation, the ABA levels were lower during pre-storage phases but higher during the storage phase compared with Bowman (Sreenivasulu *et al.*, 2010). The switch from decreased to increased ABA levels in *seg8* occurred at the end of the transition phase, at 7–9 DAF, when the shrunken-endosperm phenotype of *seg8* emerges (Supplementary Fig. 2).

The anti-ABA antibodies obtained from rabbits were characterized by competitive ELISA for two antibody fractions indicated in green and blue using ABA and jasmonic acid (Supplementary Fig. 1A). Localization and distribution of ABA was visualized in the developing NP by immunolabelling. To verify that the anti-ABA antibodies specifically recognized ABA, barley leaves were infiltrated with ABA, fixed by EDC and immunolabelled with anti-ABA antibodies (Supplementary Fig. 1B–D). Samples treated with EDC were used as a control. Immunolabelling strongly stained sections derived from ABA-infiltrated leaves (Supplementary Fig. 1B) but not from control leaves (Supplementary Fig. 1C). Pre-incubation of anti-ABA antibodies with 25mM ABA before immunolabelling diminished the green fluorescent signals from sections of infiltrated leaves (Supplementary Fig. 1D), indicating that antibodies specifically recognized ABA.

**Fig. 6.**
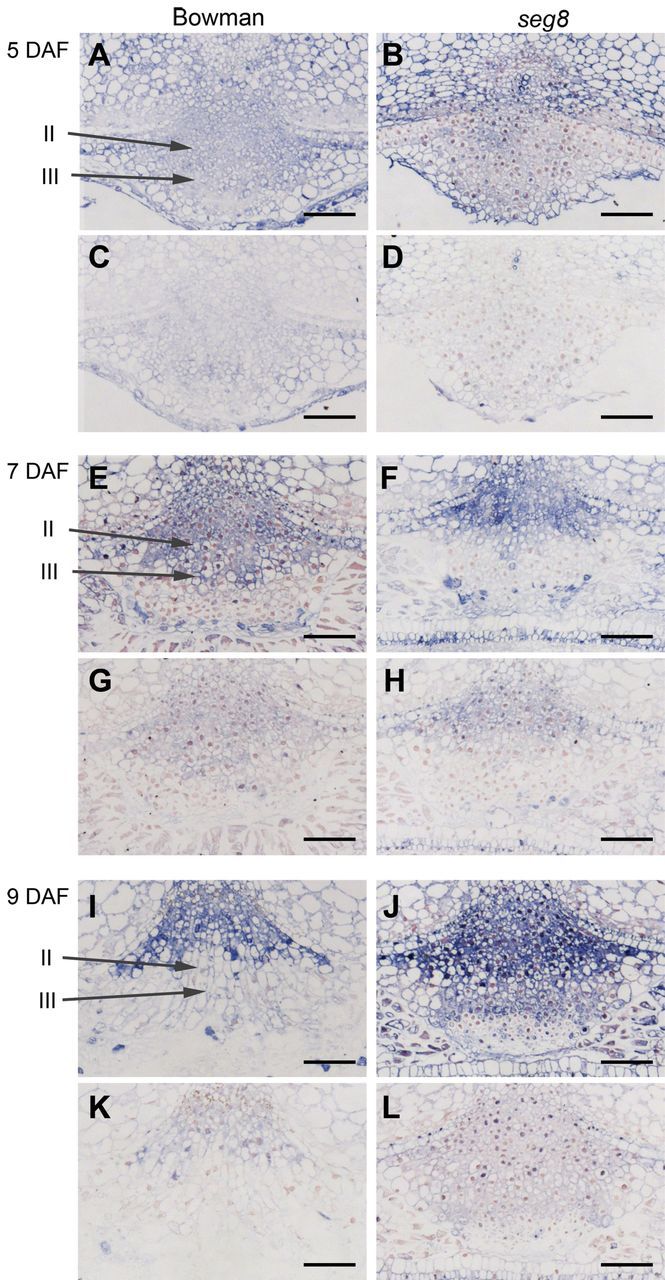
ABA distribution pattern in developing NPs of Bowman and *seg8* analysed by immunolabelling of cross-sections at 5, 7, and 9 DAF (A, B, E, F, I, J). Blue staining indicates the presence of ABA. Controls were done by pre-incubation of antibodies with ABA exhibiting very weak staining (C, D, G, H, K, L). Morphologically different cell types are indicated for immunolabelled section of Bowman NPs (II, elongated cells; III, cuboid cells with thickened cell walls). Bars, 100 µm.

At 5 DAF, blue staining indicated that ABA was present within all cells of Bowman and *seg8* NPs with a similar pattern (Fig. 6A, B). Immunostaining at 7 DAF revealed ABA presence in type II cells of Bowman but not of *seg8*. Type III cells were less labelled in the Bowman NP. The NP cells adjacent to the endosperm and nucellar epidermis in Bowman and *seg8* were strongly labelled (Fig. 6E, F). At 9 DAF, strong differences in immunostaining were detected between Bowman and *seg8* NPs. In Bowman, signals were present only in type I cells, whereas in the *seg8* NP, strong immunolabelling was detected in type I, II and III cells (Fig. 6I, J). Controls performed by pre-saturation of antibody with 25mM ABA before immunolabelling always exhibited very weak staining (Fig. 6C, D, G, H, K, L). The results indicated that, in contrast to Bowman, ABA accumulated strongly at the beginning grain filling in type II and III cells of *seg8* NPs, accompanied by substantially reduced cell elongation.

**Fig. 7.**
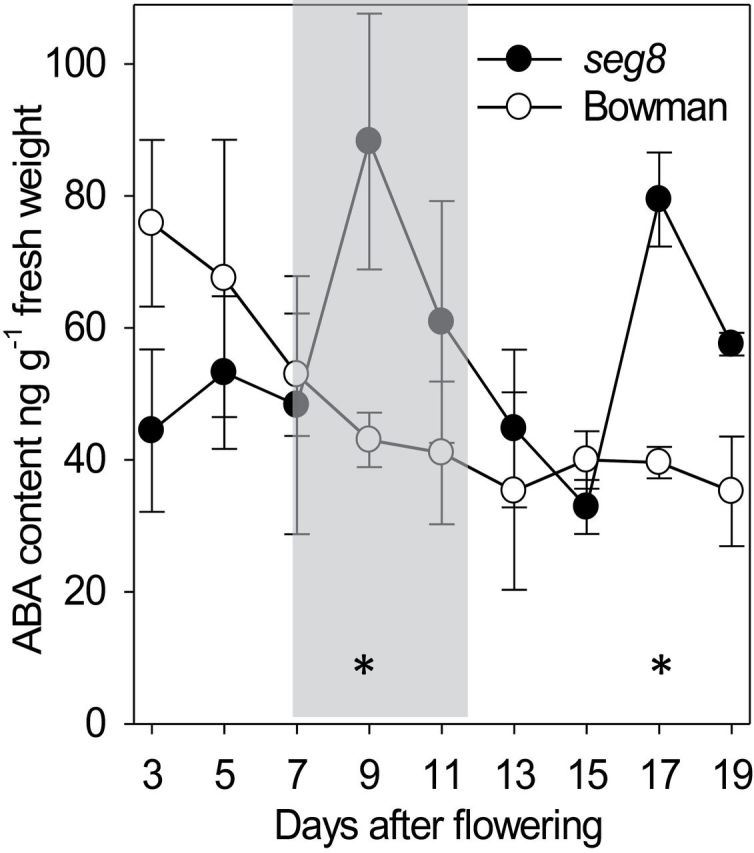
Concentrations of ABA in developing Bowman and *seg8* caryopses. The ABA content was determined between 3 and 19 DAF. Data are means±standard error (SE) of three biological replicates. Asterisks indicate significant differences according to Student’s *t*-test at *P*<0.05.

The ABA content in whole caryopses of *seg8* and Bowman was measured between 3 and 21 DAF. In Bowman, levels were highest at 3 DAF and decreased continuously to a constant level between 12 to 21 DAF. Compared with this, the ABA levels in *seg8* were lower at 3 DAF and were transiently increased between 8 and 10 DAF and showed higher levels at 17 DAF (Fig. 7).

### Measurement of GAs in *seg8* and Bowman caryopses

**Fig. 8.**
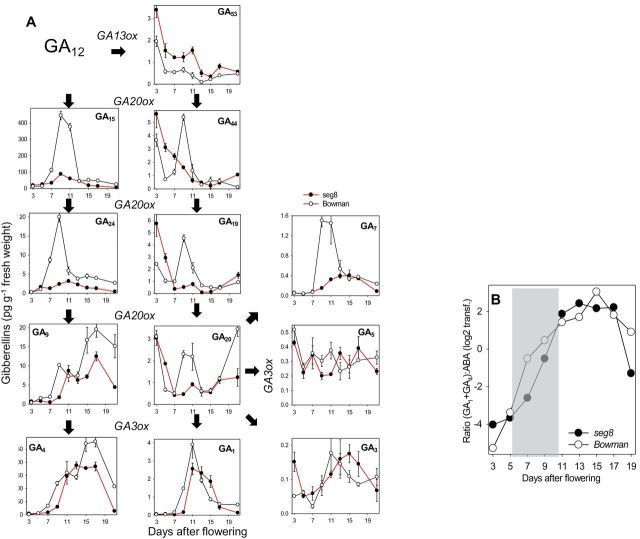
(A) Simplified GA biosynthesis pathway and concentrations of 12 GA isoforms determined in caryopses of Bowman and *seg8* between 3 and 21 DAF. Data are means of three biological replicates±SE. For statistical analysis, see Supplementary Table 1. (B) Ratio of the sum of GA_1_ and GA_4_ to ABA determined for Bowman and *seg8*. Values were calculated from data presented in Figs 7 and 8A. (This figure is available in colour at *JXB* online.)

We reported previously that GAs may be involved in generating the differentiation gradient within the NP (Thiel *et al.*, 2008). Therefore, the concentration of GA metabolites was measured in developing caryopses of *seg8* and Bowman between 3 and 21 DAF. Eighteen different GAs were detected (Supplementary Table 1 at *JXB* online). A simplified pathway of their biosynthesis including bioactive compounds GA_1_, GA_3_, GA_4_, GA_5_, and GA_7_ (Hedden and Phillips, 2000; Yamaguchi, 2008) from the precursors GA_12_ and GA_53_ is shown in Fig. 8A along with their concentrations in Bowman and *seg8* caryopses. GA_12_ and GA_53_ were oxidized in three to four steps in parallel pathways into GA_9_ and GA_20_ by GA20 oxidases (GA20oxs), the 2-oxoglutarate dependent dioxygenases (2ODDs). The formation of bioactive compounds was catalysed by a GA3 oxidase (GA3ox), another 2ODD (Hedden and Thomas, 2012). In Bowman and *seg8*, levels of the 13-non-hydroxylated gibberellins GA_15_, GA_24_, and GA_9_ and their bioactive biosynthetic product GA_4_ were very low from 3 to 5 DAF (Fig. 8A) and increased in Bowman but not in *seg8* at 7 DAF. In contrast, levels of 13-hydroxylated gibberellins GA_44_–GA_20_ in the parallel pathway, but not their bioactive forms GA_1,_ GA_5_, GA_3_, and GA_7_, were high from 3 to 5 DAF in both Bowman and *seg8* (Fig. 8A). The levels of GA_8_ were similar to those of GA_20_, which is its biosynthetic precursor. After 7 DAF, almost all GAs showed a transient increase in Bowman caryopses, which was absent or delayed (and often weaker) in *seg8* (Fig. 8A, Supplementary Table 1). Thus, in *seg8*, the levels of all intermediates from GA_44_ to GA_1_ were lower during the transition phase at 7–9 DAF compared with Bowman. It is interesting that absolute levels of GA_15_ were extremely high from 7 to 11 DAF with maxima at 9 DAF in both Bowman and *seg8*, while the content of this GA was approximately 5-fold higher in Bowman compared with *seg8* (Fig. 8A). A similar ratio between Bowman and *seg8* was found for the GA_15_ downstream metabolic products GA_24_→GA_9_→GA_4_ as well as GA_44_→GA_19_→GA_7_ (Fig. 8A). Hence, the transient GA peak, normally present during the transition stage, around 7–9 DAF, was absent or delayed in *seg8* and the GAs levels were 5–10-fold lower at that stage, whereas the level of their biosynthetic precursor GA_53_ were higher in *seg8* at all stages (Fig. 8, Supplementary Table 1). This might indicate a blocked conversion of GA_53_ to GA_44_ in *seg8*. Levels of presumed degradation products of the bioactive forms, GA_51_, GA_34_, GA_8_, and GA_29_, were significantly lower in *seg8* compared with Bowman (Supplementary Table 1). This makes it unlikely that stimulated GA degradation could cause the lower contents in *seg8.*

Taken together, the content of most GA intermediates and their bioactive forms were strongly and transiently increased in Bowman during transition and early storage phases around 7–17 DAF. In *seg8*, this increase was either missing or delayed by 2–3 d and/or much less pronounced (Fig. 8, Supplementary Table 1).

In Bowman and *seg8*, the amounts of GA and ABA behaved reciprocally with a clearly high ABA concentration in *seg8* and high GA concentrations in Bowman at the transition stage. Accordingly, in Bowman, the ratios of the sum of GA_1_ and GA_4_ to ABA were low during the pre-storage phase (3–5 DAF) followed by a strong increase during transition stage (5–11 DAF) (Fig. 8B). This inverse performance of GA and ABA suggests developmentally regulated changes in ratios of both phytohormones during regular early grain development. In *seg8*, during the transition stage, the levels of GAs were lower and the ABA levels were higher compared with Bowman. The characteristic increase in the (GA_1_+GA_4_):ABA ratio was delayed by 2–3 d with higher values at the pre-storage phase but decreased values at the transition stage (Fig. 8B).

Considering the antagonistic functions of GA and ABA, specific balances in the NP are most probably important for regulated NP differentiation during early grain development. Furthermore, altered ratios of GAs:ABA in *seg8* implicate deregulated hormone balances, which might be related to the observed developmental aberrations in this mutant.

### Expression analysis of genes involved in ABA/GA metabolism in Bowman and *seg8* NPs

The increased ABA level in *seg8* NPs suggested an induced ABA biosynthesis compared with Bowman. The rate-limiting step in ABA biosynthesis involves 9-*cis*-epoxycarotenoid dioxygenases (NCEDs), which catalyse oxidative cleavage of 9-*cis*-violaxanthin and 9-*cis*-neoxanthin into xanthoxin in plastids (Qin and Zeevaart, 2002). Xanthoxin is further converted to abscisic aldehyde, which is oxidized to ABA by aldehyde oxidases (AAOs) (Seo *et al.*, 2004).

**Fig. 9.**
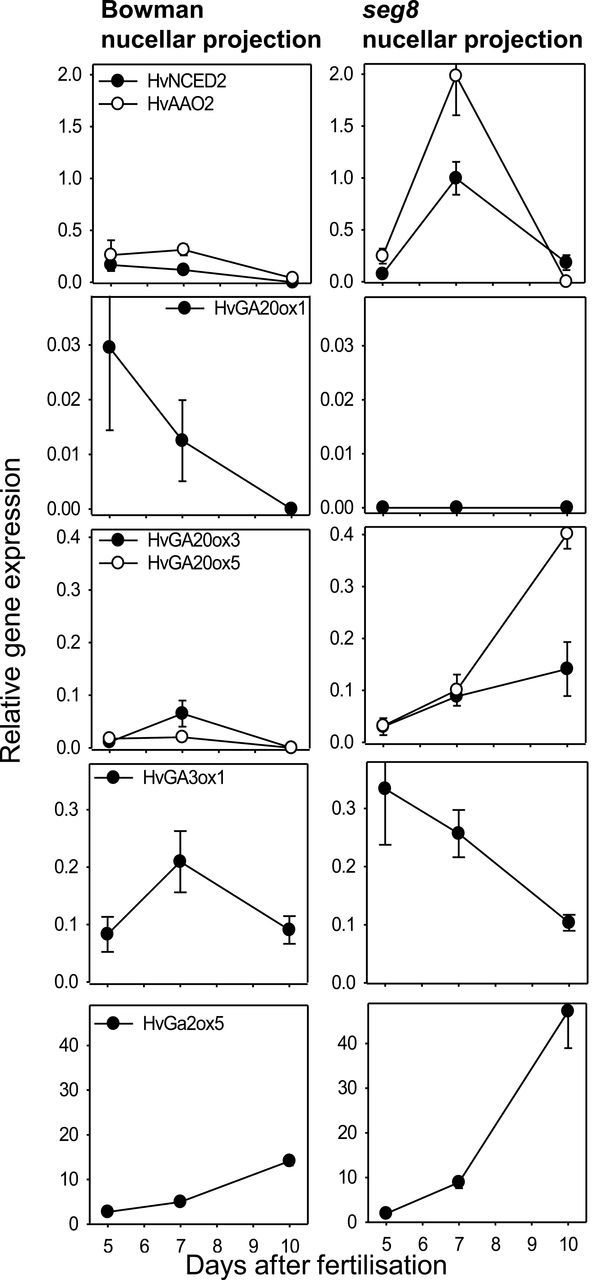
Relative expression levels of genes involved in ABA and GA metabolism in NPs of Bowman and *seg8*. Tissues were microdissected following RNA extraction and qRT-PCR. Values are means of five biological replicates±SE.

In *seg8*, higher levels of GA_53_ and lower amounts of GAs in the subsequent 13-non-hydroxylated pathway indicated blocked conversion of GA_53_ to GA_44_. GA_44_ was further oxidized at C-20 by GA20oxs (2ODDs) yielding GA_19_ and GA_20_. Bioactive GAs were then formed by further oxidation steps under the catalysis of GA3ox. The inactivation of GAs occurred by 2β-hydroxylation catalysed by GA2-oxidases (Hedden and Thomas, 2012). As ABA and GA biosynthesis enzymes NCEDs, AAOs, and GA-2ODDs are encoded by multiple genes, barley genomic resources (http://webblast.ipk-gatersleben.de/barley/viroblast.php) were screened for family members of *HvNCED*, *HvAAO*, *HvGA20ox*, *HvGA3ox*, and *HvGA2ox*. Barley contains at least four NCEDs, six AAOs, six GA20ox, two GA3ox, and eight GA2ox. Phylogenetic trees depicted these members together with those of *Arabidopsis* and rice (Supplementary Fig. 4 at *JXB* online). To monitor gene expression involved in ABA and GA biosynthesis, NPs were microdissected from Bowman and *seg8* at 5, 7, and 10 DAF following RNA isolation and qRT-PCR. From the analysed genes, only those showing significant expression and differences in transcript amounts between Bowman and *seg8* are shown in Fig. 9. Expression of the ABA biosynthesis genes *HvNCED2* and *HvAAO2* in Bowman NP slightly decreases from 5 to 7 and 10 DAF. In *seg8* NP, these genes were similarly expressed at 5 DAF but revealed around 10-fold higher mRNA levels at 7 DAF. Expression of *HvGA20ox1* decreased in Bowman NPs 3-fold from 5 to 7 DAF and further to undetectable levels at 10 DAF. In *seg8*, expression of *HvGA20ox1* was below the detection limit at all stages. The other predicted GA biosynthesis genes were similarly expressed in Bowman and *seg8* NPs at 7 DAF but elevated in *seg8* either at 5 DAF (*HvGA3ox1*) or 10 DAF (*HvGA20ox3, HvGA20ox5*, and *HvGA2ox5*) (Fig. 9). These results indicated transiently increased expression of ABA biosynthesis genes in the *seg8* NP at 7 DAF, namely of *HvNCED2* and *HvAAO2*. In contrast, the expression level of the GA biosynthesis gene *HvGA20ox1* was reduced below the detection limit in *seg8* NPs between 5 and 10 DAF. Thus, reciprocal expression of these genes could reflect the switch in the (GA_1_+GA_4_):ABA ratio during NP development.

## Discussion

In cereal grains, the NP constitutes the link between maternal and filial organs forming the transfer path for signals, assimilates and nutrients towards the endosperm. Transfer depends on the particular morphology of the NP. Hence, regular differentiation of the NP is required for proper growth and development of the endosperm (Radchuk *et al.*, 2006; Wang *et al.*, 1995; Cochrane, 1983). Moreover, the invention of a NP in Triticeae is probably important for evolution of large and round grains (Hands *et al.*, 2012). In barley grains, the NP undergoes dynamic and regulated differentiation, which is adapted to the endosperm growth. At the beginning of the storage phase, the characteristic pattern of proliferating, elongating, and disintegrating cells is established. We here have evidenced that this pattern is controlled by GA:ABA balances. When the ratios are deregulated, as in the maternally affected *seg8* mutant, differentiation of the NP is impaired, which potentially compromises signalling and transfer processes and consequently leads to aberrant endosperm growth and a flattened grain shape.

### ABA maintains the undifferentiated state preventing cell elongation in type I cells of the NP

ABA decreases growth and retards cell enlargement in many plant organs such as meristems (Barlow and Pile, 1984) and lessens intracellular swelling (Risueno *et al.*, 1971). In coffee seeds, ABA decreases the abundance of microtubules, thereby inhibiting embryo cell expansion (Da Silva *et al.*, 2008).

Proliferation of type I cells is prerequisite to replace type IV cells that are continuously disintegrating. Cell proliferation in type I regions of the NP occurred in all analysed stages in both Bowman and *seg8* (Fig. 3). Type I cells remained roundish and did not differentiate, in contrast to type II Bowman cells, which were elongating. Immunostaining of ABA revealed a distribution pattern that changed during development and differed between Bowman and *seg8* for type II and type III cells (Fig. 6). In contrast, in type I cells, ABA labelling was consistent over development and remained unchanged between Bowman and *seg8*. Thus, ABA presence in type I cells of the developing NP was related to cell proliferation and prevented cell enlargement. We therefore concluded that cell division in the *seg8* NP was not affected and therefore not involved in establishment of the mutant phenotype.

### Differentiation of the NP during the transition stage is accompanied by increasing GA:ABA ratios

Cell elongation is one of the major effects of GA in various plant organs. GA is frequently detected at high levels in growing seeds (Yamaguchi, 2008). In *Arabidopsis*, fertilization is a prerequisite for *de novo* GA biosynthesis, which in turn promotes initial elongation of siliques (Hu *et al.*, 2008). GAs produced in maternal tissues are also important for early seed development in *Arabidopsis* (Singh *et al.*, 2002), pea (Swain *et al.*, 1995), and water melon (Kang *et al.*, 1999). In pea, the early growth of seed coats and subsequent expansion of branched parenchyma cells are correlated with transcript abundance of GA biosynthesis genes and GA concentration (Nadeau *et al.*, 2011), whereas GA deficiency during the pre-storage phase disrupts embryo development in pea (Swain *et al.*, 1995). GA generally functions antagonistically to ABA. Disturbed seed development of *lh2* pea seeds is accompanied by reduced GA_1_ and GA_3_ but with higher ABA levels (Batge *et al.*, 1999).

Analysing 18 GAs including biosynthetic precursors and metabolic products in Bowman caryopses revealed transient increases in levels of most GAs including bioactive GA_1_, GA_4_, and GA_7_ between 7 and 12 DAF (Fig. 8A, Supplementary Table 1), a time at which the ABA level decreased (Fig. 7). Thus, during the transition phase (Fig. 8B, grey shaded area), a characteristic shift occurred from a low to a high (GA_1_+GA_4_):ABA ratio. In Bowman NPs, this specific shift was accompanied by elongation of type II cells. The pattern of elongated type II and type III cells with thickened walls was established at 9 DAF (Fig. 4). In *seg8* NPs, elongation of type II cells was absent at the transition stage and was only slightly shaped at 13 DAF, the beginning of the storage phase (Fig. 4). Thus, the *seg8* NP displayed a differentiation phenotype. Remarkably, at 7 DAF, ABA distribution patterns in the *seg8* NP were similar to Bowman at 9 DAF, whereas the ABA distribution in *seg8* NP at 9 DAF was similar to Bowman NP at 7 DAF (Fig. 6). This suggests that developmentally regulated gradients of ABA distribution in NPs become inverted in *seg8* during the transition phase. Furthermore, at 7 DAF, ABA was present in type I as well as type II cells of Bowman but only in type I and not in type II cells of *seg8*. However, ABA amounts were similar (Fig. 7), indicating that ABA amounts in type I cells of *seg8* were higher than in type I cells of Bowman. Possibly, ABA movement is blocked within the *seg8* NP between 5 and 7 DAF, which may reduce ABA levels in type II cells, thereby deregulating gradients of ABA distribution. Obviously, to induce elongation of type II NP cells, the presence of ABA as well as a high GA:ABA ratio is necessary. The drastic increase in ABA amounts in *seg8* at 9 DAF (Fig. 7) points to feedback regulation, which establishes in *seg8* a similar (GA_1_+GA_4_):ABA ratio as found in Bowman 2 d earlier (Fig. 8B). However, because of this spatial–temporal deregulation of GA:ABA ratios, elongation of type II cells of *seg8* NP is strongly reduced and accompanied by cell-wall thickening in both type II and type III cells and by the absence of PCD and cellular disintegration.

We showed here that differentiation of the barley NP during the transition stage is accompanied by specific shifts from lower to higher GA:ABA ratios. Against the background of well-accepted functions of phytohormones, it was concluded that spatial–temporal changes of GA:ABA balances are required for regulated development of the maternal NP. Such a scenario clearly indicates that GA:ABA balances govern early events of NP differentiation during the transition stage by analogy to the decision between germination and dormancy in mature grains after imbibition (White *et al.*, 2000). Although the genetic defect in *seg8* is as yet unknown, the mutation obviously affects adjustment of GA:ABA balances within maternal grains and/or the NP.

### Establishment of GA:ABA balances in the NP occurs by differential gene expression

GA20ox are important enzymes determining GA concentrations. In barley, the gene family consists of at least six members (Supplementary Fig. 4). In *Arabidopsis*, the members of the GA20ox family are differentially expressed and diverge in their contribution to GA biosynthesis in different organs (Rieu *et al.*, 2008). In the *seg8* NPs, *HvGA20ox1* transcripts were not detectable at any stage analysed, whereas expression of *HvGA20ox5* was higher at 5 DAF compared with Bowman (Fig. 9). However, *HvGA20ox5* and *HvGA20ox3* showed higher expression at 10 DAF, probably to compensate for the lack of GA20ox activity at 5–7 DAF.

A tight regulation of GA:ABA balances has been suggested in *Arabidopsis* seed development (Yamaguchi, 2008), probably by regulatory loops (Gazzarrini *et al.*, 2004). Accordingly, in *seg8* NPs, ABA levels increase at 9 DAF together with upregulated expression of the ABA biosynthesis genes *HvNCED2* and *HvAAO2* at 7 DAF (Fig. 9). It is as yet unknown whether the deregulated GA:ABA balance at the transition stage relies on the level of biosynthesis or signal transduction and further research is required to understand the principles of developmental changes in GA:ABA balances during NP differentiation.

### Formation of the differentiation gradient is prerequisite for optimized assimilate transfer through the NP

In contrast to Bowman, the *seg8* NP is characterized by abnormally thickened walls of type II and type III cells at 7 and 9 DAF. Type II cells fail to elongate, and cell disintegration is missing (Figs 3 and 4). In the early NP, cell-wall thickening is due to callose formation (Cochrane and Duffus, 1980). Callose deposition is induced by ABA in response to pathogenic fungi (García-Andrade *et al.*, 2011). In *seg8*, callose could overaccumulate during the transition stage due to increased ABA levels and limiting degradation as a consequence of limiting bioactive GA, which is required to induce degrading enzymes such as 1,3-β-glucanases (Rinne *et al.*, 2011). In tomato fruits, reduced maternal GA20ox1 activity impaired seed development and seed abortion is accompanied by callose formation in ovules and seed remnants at 15 DAP. This clearly indicates a sporophytic effect of limiting GAs (Olimpieri *et al.*, 2011). The presence of callose in barley NPs shortly after fertilization (Cochrane and Duffus, 1980) constitutes a plugging and isolating effect, which could protect the young endosperm against invading pathogens. Thus, in *seg8* NPs, the lower GA:ABA ratio during transition stage would favour callose deposition and inhibit its degradation. The resulting prolonged isolation of the endosperm against the NP could then prevent the transfer of signals and assimilates necessary for endosperm development.

Lacking cell disintegration in the *seg8* NP could also be related to limiting GAs. Cell death in aleurone cells of germinating wheat and barley grains is hormonally regulated and accelerated by GAs whereas it is inhibited by ABA (Bethke *et al.*, 1999). In barley aleurone cells, GA stimulates the secretion of hydrolytic enzymes, thereby triggering the onset of PCD, whereas ABA antagonizes GA effects and inhibits enzyme secretion and PCD (Fath *et al.*, 2002). Thus, analogous to the barley aleurone, attenuated cell disintegration and PCD in *seg8* NP can be explained by decreased GA:ABA ratios. Additionally, high ABA levels can exert protective effects and, for example, may detoxify reactive oxygen species, which are thought to be involved in PCD execution in a GA-dependent manner (Wu *et al.*, 2011). This indicates that a well-balanced GA:ABA ratio is necessary for establishing the differentiation gradient within the NP, which itself is prerequisite for optimized assimilate transfer through the NP. According to the potentially reduced permeability within *seg8* NPs, the monitoring of sucrose allocation reveals decreased flux within the mutant NP towards central (prismatic) endosperm (Melkus *et al.*, 2011).

The phenotype of *seg8* is somewhat reminiscent to *Jekyll*-repressed barley grains (Radchuk *et al.*, 2006). The small cysteine-rich Jekyll protein localizes within the NP and is required to properly induce PCD in the NP. In *Jekyll*-repressed grains, PCD is impaired together with sucrose release into the endosperm cavity and reduced starch accumulation in the endosperm. However, cytometric and histological studies of *Jekyll*-repressed caryopses showed that the cell number of caryopses is not different from the wild type at 4 DAF (Radchuk *et al.*, 2006). In contrast, *seg8* transfer cells, aleurone, subaleurone, and prismatic endosperm cells are substantially reduced, indicating that, besides a deficient assimilate supply, additional signals are missing, which are regularly transmitted via the NP and affect the development of endosperm regions in contrast to the NP. We conclude that, to ensure regulated differentiation and growth of the endosperm, such signals must apparently be provided at very early phases by maternal tissues. This is consistent with previous ideas that limiting amounts of ABA may cause disturbed cellularization at very early stages, thereby indicating that the *seg8* phenotype is partially caused by disturbed ABA-releasing pathways (Sreenivasulu *et al.*, 2010).

## Supplementary data

Supplementary data are available at *JXB* online.

Supplementary Fig. 1. Characterization of the anti-ABA antibodies obtained from rabbits after immunization with ABA–BSA.

Supplementary Fig. 2. Photographs taken from grains of Bowman and *seg8* at 16 DAF.

Supplementary Fig. 3. Hand sections through basal region of developing Bowman and *seg8* caryopses.

Supplementary Fig. 4. Phylogenetic trees depicting members of the ABA and GA biosynthesis gene families of barley together with those of *Arabidopsis* and rice.

Supplementary Table 1. Values of different GAs measured in caryopses of Bowman and *seg8*.

Supplementary Data

## Abbreviations:

2ODD: 2-oxoglutarate dependent dioxygenase
AAO: aldehyde oxidase
ABA: abscisic acid
DAF: days after flowering
EDC: 1-ethyl-3-(3-dimethylaminopropyl) carbodiimide
GA: gibberellin
NCED: 9-cis-epoxycarotenoid dioxygenase
NP: nucellar projection
PCD: programmed cell death
qRT-PCR: quantitative reverse transcription-PCR
SE: standard error
TUNEL: terminal deoxynucleotidyl transferase dUTP nick end labelling.
