# Supplementary material for: Gibberellin-to-abscisic acid balances govern development and differentiation of the nucellar projection of barley grains
Source: J Exp Bot. 2014 Jul 14;65(18):5291–304. doi: 10.1093/jxb/eru289 (PMC4157710; doi:10.1093/jxb/eru289)
Supplement: Supplementary Data [file supp_65_18_5291__index.html]

Gibberellin-to-abscisic acid balances govern development and differentiation of the nucellar projection of barley grains — Gibberellin-to-abscisic acid balances govern development and differentiation of the nucellar projection of barley grains — Supplementary Data 

# Gibberellin-to-abscisic acid balances govern development and differentiation of the nucellar projection of barley grains

## Supplementary Data

Data files

**Files in this Data Supplement:**

- Supplementary Data - Supplementary Data
