# Supplementary material for: Gibberellin-to-abscisic acid balances govern development and differentiation of the nucellar projection of barley grains
Source: J Exp Bot. 2014 Jul 14;65(18):5291–304. doi: 10.1093/jxb/eru289 (PMC4157710; doi:10.1093/jxb/eru289)
Supplement: Supplementary Data [file supp_eru289_jexbot121889_file001.pdf]

Table S1, Values of the different GA isoforms measured in caryopses of Bowman and *seg8* . Values are in ng g^{-1} fresh weight and
and are means of 3 biological replicates. Significant difference according to *t* -test, red and green significantly decreased and increased in *seg8* respectively.

seg8	GA15		GA24		GA19		GA20		GA1		GA7		GA5		GA3		GA44		GA9		GA4		GA53		GA6		GA8		GA13		GA29		GA34		GA51	
**DAF**	**Mean**	**SD**	**Mean**	**SD**	**Mean**	**SD**	**Mean**	**SD**	**Mean**	**SD**	**Mean**	**SD**	**Mean**	**SD**	**Mean**	**SD**	**Mean**	**SD**	**Mean**	**SD**	**Mean**	**SD**	**Mean**	**SD**	**Mean**	**SD**	**Mean**	**SD**	**Mean**	**SD**	**Mean**	**SD**	**Mean**	**SD**	**Mean**	**SD**
**3**	**19.55**	2.91	0.26	0.01	**5.74**	1.05	3.04	0.34	0.05	0.01	0.06	0.01	0.43	0.02	0.15	0.03	5.62	1.03	**0.70**	0.13	4.60	0.60	**3.39**	0.35	0.15	0.01	2.81	0.33	0.25	0.04	0.60	0.10	**1.09**	0.08	6.63	1.35
**5**	**25.30**	3.60	**1.63**	0.11	**2.93**	0.35	**1.86**	0.03	0.06	0.01	0.03	0.00	0.22	0.04	**0.05**	0.01	**3.11**	0.31	**0.78**	0.18	5.29	0.30	**1.53**	0.32	0.13	0.04	0.99	0.16	**0.13**	0.03	0.40	0.11	**0.60**	0.04	5.29	1.43
**7**	**33.87**	3.27	**1.27**	0.20	**0.36**	0.05	0.43	0.07	**0.02**	0.01	0.08	0.01	0.34	0.06	0.06	0.02	**2.45**	0.23	**0.47**	0.07	**8.69**	0.72	**1.21**	0.05	0.13	0.03	**1.47**	0.04	**0.11**	0.02	0.18	0.05	**1.08**	0.10	**2.53**	0.14
**9**	**88.19**	0.82	**2.39**	0.21	**0.52**	0.07	**0.47**	0.06	**0.16**	0.05	**0.15**	0.02	**0.20**	0.02	0.08	0.03	**1.61**	0.11	**1.77**	0.36	**30.10**	1.12	**1.23**	0.21	0.11	0.03	**1.41**	0.10	0.26	0.05	**0.02**	0.00	**1.34**	0.08	**2.64**	0.53
**11**	**60.41**	1.69	**3.17**	0.35	**0.77**	0.14	0.92	0.08	2.56	0.32	**0.32**	0.01	**0.21**	0.01	0.11	0.01	0.64	0.16	8.71	1.44	147.08	1.70	**1.54**	0.16	0.16	0.02	**0.77**	0.11	0.56	0.10	**0.08**	0.02	**3.54**	0.18	**2.65**	0.35
**13**	44.72	1.60	**2.25**	0.29	**0.08**	0.02	0.58	0.20	2.32	0.35	0.39	0.04	0.36	0.04	0.16	0.06	0.47	0.07	6.26	0.85	**187.96**	13.70	**0.50**	0.14	0.18	0.01	**1.75**	0.21	2.02	1.17	**0.23**	0.08	**4.71**	0.57	**2.96**	0.22
**15**	**18.74**	1.14	**1.43**	0.16	**0.13**	0.01	0.55	0.05	**1.86**	0.32	0.40	0.11	0.27	0.04	0.17	0.03	0.23	0.18	**7.22**	0.53	**177.26**	1.71	0.35	0.04	0.16	0.01	**2.79**	0.79	**7.09**	1.52	**0.09**	0.02	**4.20**	0.40	**0.80**	0.32
**19**	**14.74**	1.15	**1.28**	0.21	0.53	0.04	1.12	0.16	0.44	0.14	0.34	0.03	0.39	0.08	0.15	0.04	0.45	0.03	**12.50**	1.19	**183.95**	16.68	**0.80**	0.12	**0.18**	0.03	**6.86**	0.47	**9.65**	0.60	0.45	0.01	**41.08**	0.80	**1.70**	0.32
**21**	**6.63**	0.13	**0.43**	0.03	1.50	0.24	**1.24**	0.40	**0.13**	0.03	**0.09**	0.02	0.23	0.02	0.07	0.03	**1.06**	0.06	**4.40**	0.22	**14.24**	1.35	0.56	0.02	0.23	0.09	**11.43**	0.80	**9.02**	0.75	0.49	0.17	128.95	13.22	**0.86**	0.10

Bowman	GA15		GA24		GA19		GA20		GA1		GA7		GA5		GA3		GA44		GA9		GA4		GA53		GA6		GA8		GA13		GA29		GA34		GA51	
**DAF**	**Mean**	**SD**	**Mean**	**SD**	**Mean**	**SD**	**Mean**	**SD**	**Mean**	**SD**	**Mean**	**SD**	**Mean**	**SD**	**Mean**	**SD**	**Mean**	**SD**	**Mean**	**SD**	**Mean**	**SD**	**Mean**	**Mean**	**SD**	**Mean**	**SD**	**Mean**	**SD**	**Mean**	**SD**	**Mean**	**SD**	**Mean**	**SD**	
**3**	12.85	0.31	0.25	0.00	2.42	0.03	3.11	0.12	0.03	0.01	0.05	0.01	0.52	0.09	0.05	0.00	3.67	0.46	0.38	0.03	1.12	0.25	1.95	0.24	0.14	0.03	2.25	0.17	0.32	0.10	0.42	0.05	0.08	0.01	2.86	0.37
**5**	14.50	0.86	1.02	0.09	1.04	0.15	0.67	0.08	0.04	0.00	0.04	0.00	0.26	0.03	0.06	0.01	0.71	0.05	1.55	0.23	5.08	0.80	0.57	0.08	0.15	0.04	1.38	0.07	0.38	0.08	0.26	0.04	1.95	0.04	3.77	0.12
**7**	111.89	9.82	8.69	0.69	0.99	0.05	0.50	0.09	0.15	0.03	0.07	0.01	0.36	0.11	0.02	0.01	1.27	0.21	1.87	0.26	33.74	1.18	0.54	0.07	0.17	0.02	1.52	0.23	0.22	0.04	0.11	0.02	11.99	0.60	4.71	0.60
**9**	445.20	25.92	20.01	1.31	4.54	0.28	2.31	0.16	1.46	0.01	1.50	0.12	0.30	0.04	0.08	0.01	5.38	0.25	10.20	0.13	119.97	7.79	0.65	0.11	0.15	0.02	4.66	0.22	0.35	0.02	0.39	0.03	42.63	1.34	17.93	1.34
**11**	376.10	21.14	5.87	1.01	2.11	0.43	2.19	0.62	3.89	0.74	1.45	0.42	0.37	0.06	0.18	0.06	1.37	0.49	7.30	1.41	159.97	43.19	0.39	0.14	0.26	0.08	11.40	3.98	0.50	0.19	0.97	0.13	46.27	12.74	18.76	7.29
**13**	44.08	2.09	3.81	0.39	0.65	0.07	0.61	0.08	1.92	0.13	0.54	0.17	0.23	0.06	0.15	0.03	0.31	0.01	7.44	1.02	143.13	9.03	0.09	0.01	0.23	0.04	12.60	0.65	0.44	0.07	0.81	0.14	39.68	1.13	7.57	1.79
**15**	51.70	3.66	4.49	0.57	0.50	0.07	0.63	0.12	0.87	0.08	0.34	0.01	0.26	0.08	0.11	0.02	0.62	0.26	16.73	3.38	270.64	29.51	0.22	0.05	0.23	0.04	13.65	1.24	0.60	0.12	0.92	0.11	55.28	5.99	7.25	0.96
**19**	47.24	6.10	3.96	0.21	0.45	0.08	1.18	0.11	0.60	0.06	0.37	0.01	0.31	0.01	0.09	0.01	0.57	0.21	19.55	2.04	278.63	20.32	0.40	0.03	0.27	0.02	11.79	1.06	0.65	0.09	0.41	0.17	64.36	1.55	6.26	1.68
**21**	24.73	3.28	2.68	0.24	0.92	0.06	3.44	0.33	0.57	0.01	0.23	0.02	0.33	0.05	0.11	0.02	0.14	0.03	15.16	3.03	107.34	4.07	0.47	0.06	0.33	0.07	29.89	1.52	0.70	0.02	0.95	0.12	146.14	12.00	7.19	1.10

T-test, P values

T-Test DAF	GA15	GA24	GA19	GA20	GA1	GA7	GA5	GA3	GA44	GA9	GA4	GA53	GA6	GA8	GA13	GA29	GA34	GA51
**3**	0.038	0.537	0.014	0.827	0.081	0.760	0.292	0.012	0.087	0.047	0.002	0.013	0.677	0.119	0.493	0.091	0.000	0.033
**5**	0.021	0.005	0.004	0.000	0.145	0.062	0.361	0.223	0.001	0.027	0.760	0.018	0.600	0.046	0.031	0.182	0.000	0.278
**7**	0.001	0.000	0.000	0.435	0.007	0.426	0.908	0.072	0.009	0.003	0.000	0.001	0.246	0.787	0.035	0.179	0.000	0.015
**9**	0.000	0.000	0.000	0.000	0.000	0.000	0.048	0.965	0.000	0.000	0.000	0.033	0.251	0.000	0.082	0.000	0.000	0.000
**11**	0.000	0.032	0.018	0.070	0.103	0.031	0.021	0.246	0.131	0.429	0.715	0.003	0.164	0.024	0.736	0.001	0.012	0.040
**13**	0.765	0.013	0.001	0.867	0.228	0.360	0.097	0.794	0.054	0.309	0.023	0.025	0.131	0.000	0.147	0.013	0.000	0.038
**15**	0.000	0.002	0.003	0.449	0.022	0.499	0.965	0.055	0.182	0.021	0.015	0.074	0.097	0.001	0.007	0.001	0.000	0.001
**19**	0.003	0.000	0.340	0.704	0.225	0.332	0.251	0.105	0.506	0.021	0.011	0.011	0.028	0.005	0.000	0.780	0.000	0.026
**21**	0.002	0.000	0.053	0.005	0.000	0.003	0.064	0.259	0.000	0.013	0.000	0.138	0.356	0.000	0.000	0.047	0.289	0.002

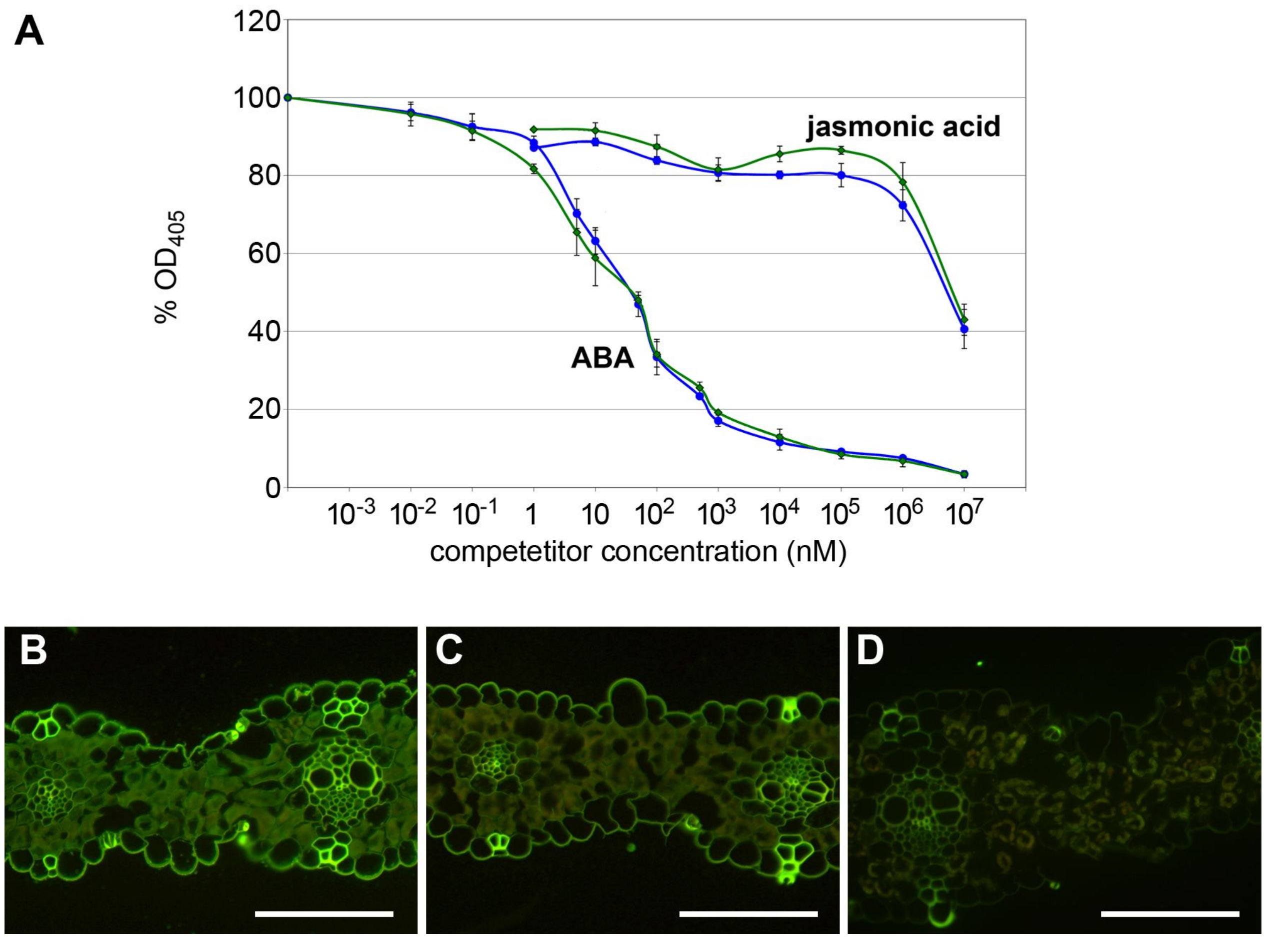

Supplementary Fig. 1. Characterization of the anti-ABA antibodies obtained from rabbits after immunization with ABA-BSA. (A) Competitive enzyme-linked immuno-sorbent assay (ELISA) for two antibody fractions indicated in green and blue using ABA and jasmonic acid. Antibodies were diluted 1:50,000 (blue) and 1:25,000 (green) and as antigen ABA-BSA was used in an amount of 0.025 µg per well. The progression of the curves for ABA envisages the binding of the antibodies to this antigen, whereas jasmonic acid is not bound by the antibodies at physiological concentrations. Values are given as means ± SD (n = 15).

(B to D) Immuno-labelling of ABA in barley leaves. Primary leaves of seedlings were infiltrated with 100 mM ABA, fixed with EDC and embedded in PEG. Immuno-localisation was performed with anti-ABA antibodies and a secondary antibody coupled with AlexaFluor488 (green fluorescence). (B) Strong green fluorescence indicates the presence of infiltrated ABA in parenchyma cells. (C) Leaves without ABA infiltration exhibit brownish colour of the parenchyma cells. Note that stomata and vascular tissues show auto-fluorescence. (D) Pre-incubation of the anti-ABA-antibody with ABA abolishes the fluorescence signal. Bars = 100 µm.

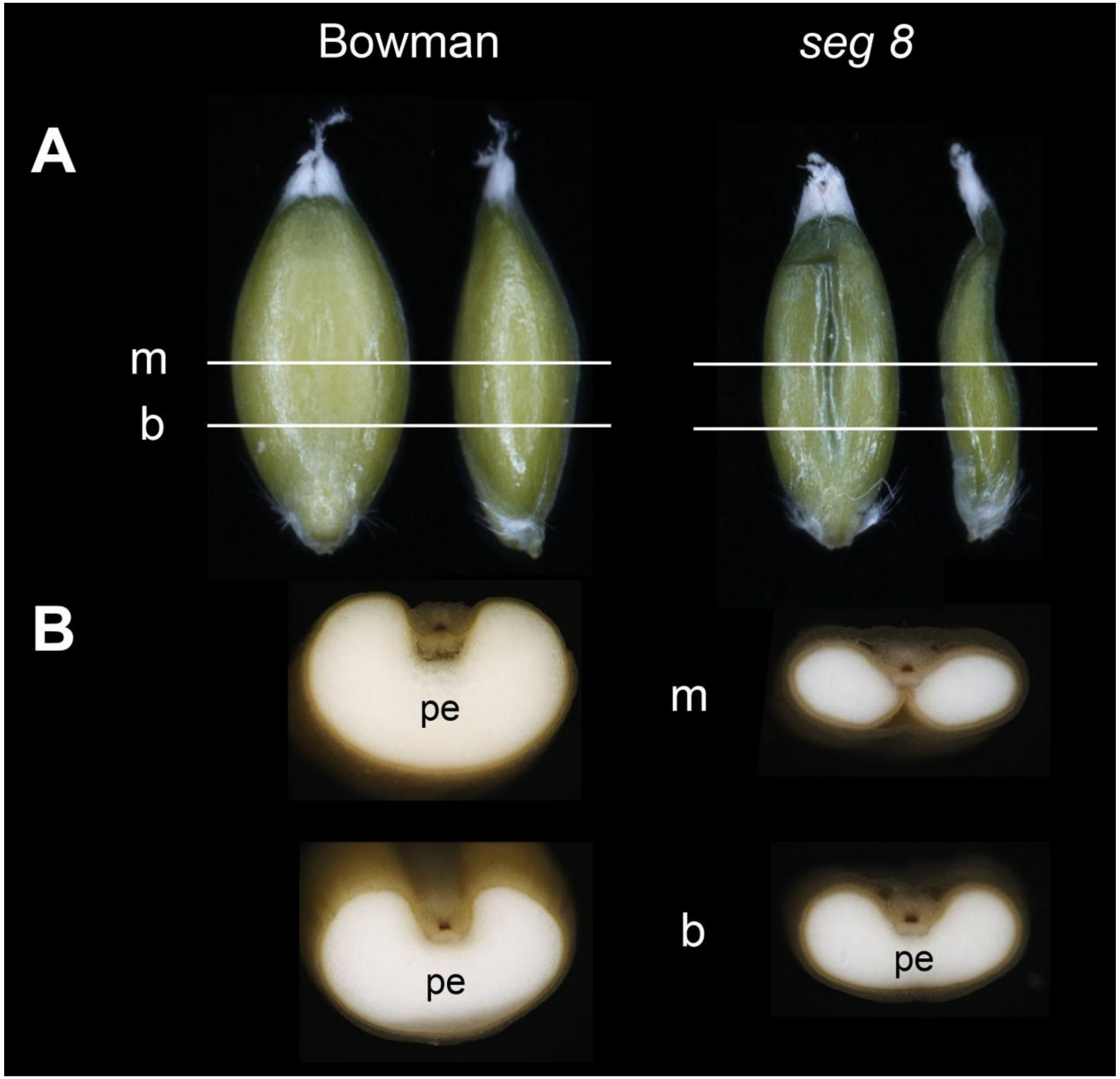

Supplementary Fig. 2. Photographs taken from grains of Bowman (top left) and *seg8* (top right) at 16 DAF. The cutting sites (m = mid, b = basal) are marked. Note that from 10 DAF the prismatic endosperm (pe) develops only in the basal area, which leads to a flattened appearance of the *seg8* grain.

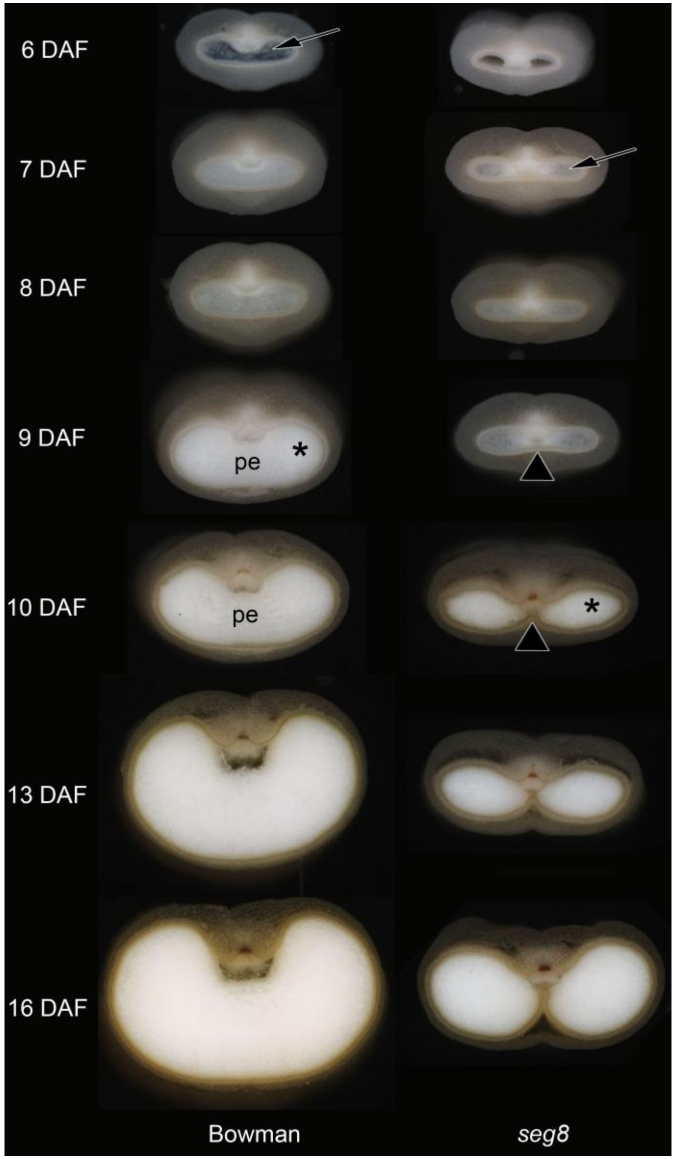

Supplementary Fig. 3. Hand sections through basal region of developing Bowman and *seg8* caryopses. The endosperm is cellularised at 6 DAF in Bowman and at 7 DAF in *seg8* (arrows). The *seg8* NP adjoins the integuments at the dorsal side during cellularisation and the prismatic endosperm is absent (arrow heads). Starch accumulation occurs from 9 DAF in Bowman and from 10 DAF in *seg8* (asterisks) as visible by the white colour of the endosperm.

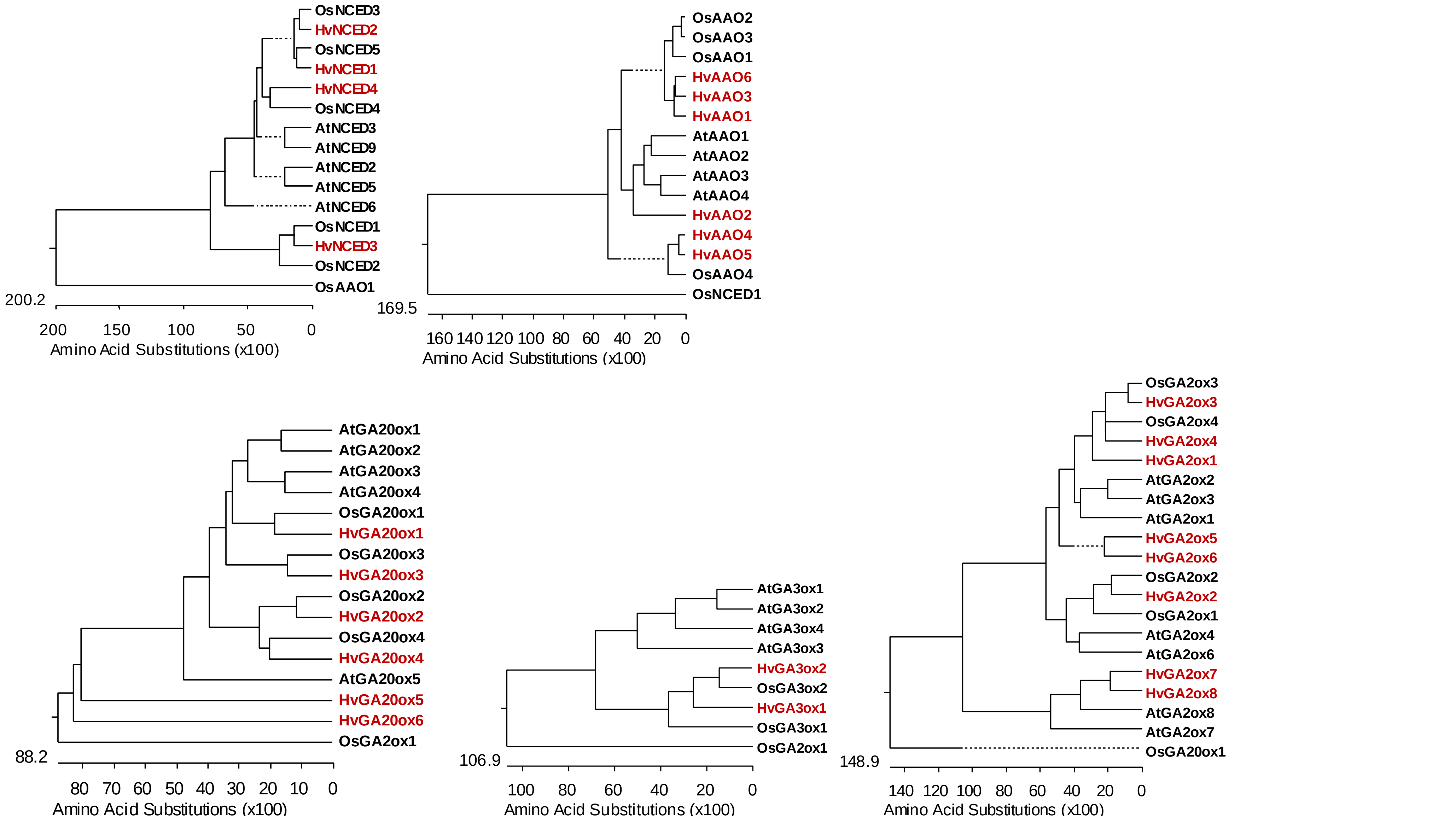

Supplementary Fig. 4. Phylogenetic trees depicting members of ABA and GA biosynthesis gene families of barley (in red) together with those of Arabidopsis and rice. Basic alignments for tree construction and corresponding sequence distance matrices were calculated using the ClustalW algorithm with BLOSUM protein weight matrix including one sequence as outgroup. All calculations and visualisations were done with Lasergene 8 (DNAStar, www.dnastar.com).
